# The Viral Mimetic Polyinosinic:Polycytidylic Acid Alters the Growth Characteristics of Small Intestinal and Colonic Crypt Cultures

**DOI:** 10.1371/journal.pone.0138531

**Published:** 2015-09-28

**Authors:** Julie M. Davies, Rebeca Santaolalla, Richard J. von Furstenberg, Susan J. Henning, Maria T. Abreu

**Affiliations:** 1 Miller School of Medicine, Division of Gastroenterology, University of Miami, Miami, Florida, United States of America; 2 Department of Medicine and Cell Biology and Physiology, University of North Carolina at Chapel Hill, Chapel Hill, North Carolina, United States of America; Indiana University School of Medicine, UNITED STATES

## Abstract

**Background & Aims:**

The intestinal epithelium is the first line of defense against enteric pathogens. We investigated the response of small intestinal and colonic crypt cultures to a panel of toll-like receptor ligands to assess the impact of microbial pattern recognition on epithelial growth.

**Methods:**

Primary murine jejunal enteroids and colonoids were cultured with lipopeptide Pam3CSK4, lipopolysaccharide (LPS) or polyinosinic:polycytidylic acid (Poly I:C) for 4 to 6 days. Surface area, budding and survival were assessed. Proliferation and numbers of lysozyme positive cells were quantified by flow cytometry. Gene expression was assessed by Nanostring and qRT-PCR.

**Results:**

Exposure to Pam3CSK4 and LPS had minimal impact on either enteroids or colonoids. In contrast, Poly I:C increased the surface area of enteroids, while colonoids demonstrated decreased budding. Survival was decreased by Poly I:C in enteroids but not in colonoids. Both enteroids and colonoids exhibited upregulated gene expression of chemokines, but these were increased in magnitude in enteroids. Decreases in gene expression associated with epithelial differentiation and lysozyme positive cells were more apparent in enteroids than in colonoids. Baseline gene expression between enteroids and colonoids differed markedly in levels of stem cell and inflammatory markers. The changes in morphology induced by Poly I:C were mediated by the toll-like receptor adaptor molecule 1 (Ticam1) in enteroids but not in colonoids.

**Conclusions:**

Poly I:C alters the molecular program of epithelial cells and shifts from absorption and digestion towards defense and inflammation. Diversity of responses to microbial patterns in enteroids and colonoids may underlie differences in susceptibility to infection along the intestinal tract.

## Introduction

The intestinal environment is home to a vast diversity of bacteria and viruses to which the immune system is largely tolerant. The symbiotic relationship that exists between the host and luminal microbes is now well recognized. Studies in germ-free mice have demonstrated that intestinal epithelial cell proliferation is decreased compared with conventionalized mice suggesting that microbial products regulate epithelial growth [1–4].

Microbial products are largely recognized by germ-line encoded innate immune sensors like toll-like receptors (TLRs). TLRs recognize a variety of microbial structures including bacterial and viral products. TLR1/2 recognizes bacterial lipopeptide Pam3CSK4, TLR4 recognizes bacterial lipopolysaccharide (LPS) and TLR3 recognizes double-stranded RNA (dsRNA) motifs, mostly associated with viruses. TLR expression in epithelial cells is detectable along the proximal to distal axis in the small intestine and colon [5]. We and others have described that TLRs are important regulators of intestinal homeostasis and are required for epithelial repair following injury [6–8]. However, in disease states including Crohn’s disease and ulcerative colitis, up-regulation of TLRs has been documented in epithelial cells and may contribute to disease pathology [9, 10]. Loss of secretory goblet cells and villus atrophy are hallmarks of rotavirus infection in the small intestine [11], and infection of epithelial cells lines with virus has been shown to upregulate TLR2, 3, 7 and 8 [12]. These studies led us to hypothesize that microbial signaling regulates stem cell biology and epithelial differentiation.

New methods to culture primary epithelial cells from human and mouse intestinal tissues have emerged as an exciting approach to study the function of the small intestine and colon epithelium [13–15]. This methodological advance has improved our ability to investigate normal epithelial responses without the confounding effects inherent to cancer cell lines. Enteroid cultures are faithful to their site of origin, as region specific function is maintained in enteroids cultured from the duodenum, jejunum and ileum [14]. Paneth cells potently increase the ability of small intestinal stem cells to grow in Matrigel cultures [16], which emphasizes the support that other niche cells provide to sustain stem cell growth. While there are no discernable Paneth cells in the colon, secretory cells found at the crypt base are in contact with neighboring stem cells and also support stem cell growth in organoids [17]. These cells are hypothesized to perform the complementary function of Paneth cells in the small intestine. Enteroids and colonoids allow the differentiation of all the epithelial cell lineages and as such, are an advantageous model for investigating the impact of exogenous stimulation on different epithelial cell subsets and on epithelial homeostasis [18].

The function of the small intestine compared to the colon is very different, as is the bacterial load, susceptibility to infection and disease and development of cancer. Epithelial pathology associated with most intestinal viruses is usually constrained to the small intestine, although the reason for this difference remains unclear. In the current study, we used jejunal and colon crypt cultures to investigate how the small and large intestine differ in their response to microbial product stimulation. Neither LPS nor Pam3CSK4 induced discernable changes in either enteroids or colonoids. We found that Poly I:C altered the morphology of both enteroids and colonoids, although with important differences. A reduction in lysozyme positive cells and expression of epithelial differentiation markers was observed in enteroids and to a lesser extent in colonoids. Both sites induced a robust inflammatory response characterized by increased gene expression of chemokines. However, survival and gene expression of stem cell markers was reduced in enteroids but not colonoids. Additionally, we found that the baseline expression of inflammatory and stem cell-associated genes varied markedly between enteroids and colonoids. Enteroid responses to Poly I:C were mediated through toll-like receptor adaptor molecule 1 (TRIF), but not colonoid responses. Collectively, our data indicate that both small intestinal and colonic stem cells respond to dsRNA but with a diverse molecular and morphologic phenotype. Our data has implications for understanding the diversity of responses of stem and progenitor cells to pathogens along the gut axis and their potential role in the susceptibility of each site to pathogenesis.

## Methods

### Mice

All studies using mice were approved by and performed according to the University of Miami Institutional Animal Care and Use Committees’ (IACUC) guidelines (Protocol number 14–041). Animal sacrifice was performed using isoflurane anesthesia followed by cervical dislocation. Wild-type (C57BL/6J) were originally purchased from Jackson Laboratories (Bar Harbor ME) and bred in-house. Ticam1^Lps2^ (C57BL/6J-*Ticam1* ^*Lps2*^/J, Jackson laboratories, Bar Harbor ME) tissue was a generous gift from Dr. Masayuki Fukata, University of California Los Angeles. Enteroids were grown from mice between 5–12 weeks old. Colonoids were grown from mice 5–8 weeks old.

### Cell culture

#### Murine enteroids

Crypts from the jejunum were isolated immediately after sacrifice as in [19] with a few modifications. Jejunum was isolated, rinsed with 10mL HBSS (Corning Cellgro, cat# 21-022-CV) cut open longitudinally and gently agitated in 10mL cold HBSS to remove remaining contents. The jejunum was then cut into 1cm pieces and transferred to 3mM EDTA (Sigma, cat# E5134-250G) in HBSS on ice for 30 min with agitation. Tissue pieces were transferred to HBSS and shaken for 3 min. The supernatant was passed through a 70μm filter and intact crypts counted. An appropriate number of crypts were centrifuged at 150g for 5 min. Crypts were resuspended in basement membrane matrix growth-factor reduced, phenol red free Matrigel (Corning, cat# 356231) and 10μL plated into 24 or 48 well dishes. Media (DMEM-F12 with glutamine (ATCC, 30–2006)) contained: B-27 (50X Life Technologies cat# 17504044), N-2 (100X Life Technologies cat# 17502–048) penicillin/streptomycin (100X Gibco, cat#15140–122), HEPES (100mM, Amresco, cat# J848-100ML), gentamicin/amphotericin (500X Life Technologies, cat# J0-0640), EGF (50ng/mL, Life Technologies, cat# PMG8045), Noggin (100ng/mL, Peprotech, cat# 250–38), human R-spondin1 (500ng/mL, Peprotech, cat# 120–38), Y27632 (10μM, Tocris: R&D Systems cat# 1254) and Chir99021 (2.5μM, Stemgent, cat# 04-0004-02).

#### Murine colonoids

Colons were excised, washed, opened longitudinally and placed in 10mL HBSS with penicillin/streptomycin and Thiazovivin (2.5μM, Stemgent, cat# 04–0017) at 4°C overnight. The next morning, colons were washed in cold HBSS and placed in 60mM EDTA in HBSS for 30 min on ice with agitation. Colons were removed to a petri dish and scraped to remove the epithelium and triturated 15 times prior to filtration through a 40μm filter. By this method the top of the crypts break apart but many of the bases have already formed spheres [20]. These were counted, and an appropriate number were centrifuged at 800g for 5 min. Crypt bases were resuspended in Matrigel and 10μL was plated into 24 or 48 well plates. Media was similar to the enteroid culture with a few differences: Jag-1 (1μM, Anaspec, cat# 61298) was included and Thiazovivin (2.5μM) was added in place of Y27632. Chir99021 was used at 5μM. Media was refreshed by replacing two-thirds of the media on day 4/5 of an 8 day culture.

#### Stimulations

Crypts were plated and allowed to grow without stimulation for two days prior to addition of stimulants. Pam3CSK4 (400ng/mL, Invivogen, cat# tlrl-pms), Poly I:C (40μg/mL, Invivogen, cat# tlrl-picw) and LPS (13μg/mL, Invivogen, cat# tlrl-pelps) were added to the media. Stimulations were repeated with the replacement of the media on day 4 of enteroid culture, and on day 4 and 6 of colonoid culture.

### Gene expression analysis

#### Nanostring analysis

Triplicate wells from enteroid and colonoid cultures were pooled and RNA extracted with RNAbee (Tel-test, cat# CS501B) using a standard protocol. 100ng of RNA was used as template for Nanostring probe based RNA analysis using a custom nCounter probe set provided by the manufacturer (**S1 Table**). Samples were run and analyzed as per the manufacturer’s instructions (Nanostring Technologies, Seattle WA). Sample counts were normalized using the nSolver 2 2.0.72 software provided by the manufacturer using background subtraction of the geometric mean of the negative controls and the geometric mean of both the positive controls and the indicated housekeeper genes (Actb, Gusb, Gapdh, Tbp) to build ratios.

#### *qRT-PCR*

Complimentary DNA (cDNA) was prepared from RNA isolated for the Nanostring analysis. 500ng of template RNA from enteroids and colonoids was converted to cDNA using random hexamers as per manufacturers’ instructions (Transcriptor First strand cDNA synthesis kit, Roche, cat# 04 379 012 001). qRT-PCR samples were run on a Lightcylcer 480 (Roche) using SybrGreen (Takara/Clontech, cat# RR420A) with the following cycling conditions: 95°C 5min followed by 50 cycles of 95°C for 10s, 58°C for 15s, 72°C for 15s and finished with 72°C for 1min. Fold change was determined using the 2^-ddCT^ method as per [21]. Primer lists are included in the supplementary section (**S2 Table**).

### Imaging

#### Area and bud count

Images used to determine area and bud count were taken using the 4X objective on a Nikon Eclipse TS100 inverted microscope with a Nikon DS-Ri1 camera. Four to five areas of interest in each well were imaged at multiple depths of focus and the number of buds on each enteroid or colonoid counted. Area was determined by drawing a freehand circumference using the NIS-Elements analysis program.

#### EdU staining and imaging

Enteroids and colonoids were embedded in Matrigel in eight well chamber slides (Lab-Tek, ThermoScientific cat# 177402). At harvest, EdU was added to culture for 50 min. Wells were then washed three times with PBS and fixed with methanol for 20 min. Following fixation, wells were then washed 3X with 3% BSA (Sigma, cat# A7906-500G) in PBS. Cells were further permeabilized with 0.5% Triton X-100 (Sigma, cat# T9284-100ML) in PBS for 40 min and then washed 3X with 3%BSA-PBS. EdU incorporation was detected as per the manufacturer’s instructions (ClickiT Plus EdU Imaging kit, Life Technologies, cat# C10339) and then counterstained with 4',6-diamidino-2-phenylindole (DAPI, 1:5000 Molecular probes, cat# D3571) for 10 min. Slides were washed 3X with 3% BSA-PBS and mounted using Slowfade Gold antifade reagent (Life Technologies, cat# S36936). Images were obtained using either the 10X (enteroid) or 20X (colonoid) objective.

#### Lysozyme staining and imaging

Enteroids and colonoids were grown in 24 well culture dishes. At harvest, wells were washed with PBS and then fixed with 4% paraformaldehyde for 20 minutes at room temperature. The organoids were harvested to an eppendorf tube and spun at 100g for 2 minutes. After the supernatant was removed they were gently resuspended and washed 2X in 3% BSA-PBS prior to transfer to a glass slide, and dried at 50°C for 20 minutes. Slides were then washed and stained with lysozyme (1:10 rabbit polyclonal anti-human Lyz1 FITC, Dako, cat# F037201-1, lot# 00095833) for 2 hrs in 0.5%TritonX-100 in PBS at room temperature, washed three times and then counterstained with DAPI and mounted and imaged as above using the 20X objective.

### Flow cytometry

#### Lysozyme and EdU staining of dissociated enteroids and colonoids

At the end of culture, 10μM EdU was added to the media and incubated for 50 min at 37°C. After incubation the media was removed and wells were washed with HBSS. The Matrigel was dissociated with 0.25% Trypsin + 0.5mM EDTA in HBSS for 2 min at 37°C. Excess DMEM-F12 was added to the wells and then harvested to a 50mL conical vial. The organoids were centrifuged and resuspended in 10mL of 3mM EDTA in HBSS (enteroids) or 60mM EDTA in HBSS (colonoids) for 20 min on ice with gentle shaking, then rendered to a single cell suspension with pipetting and filtered through a 40μm filter. Cells were washed twice with HBSS. Viability stain was added at 1:1000 (Fixable viability dye eFluor 660, eBiosciences, cat# 65-0864-14) in HBSS for 30 min on ice in the dark. Cells were then fixed with 4% paraformaldehyde in PBS for 20 min at room temperature in the dark. Following two washes in 1% BSA-PBS cells were concurrently permeabilized (0.5% TritonX100) and stained with Lysozyme antibody (1:10) for 30 min at room temperature [22]. Cells were washed and then stained with EdU as per the manufacturers’ instructions. Flow cytometric analysis was performed on a Becton Dickinson Fortessa and analyzed by FlowJo vX.0.7 software (Treestar Inc).

### Data analysis

Statistical analyses were performed using the GraphPad Prism 6.04 software for Windows (GraphPad Software, La Jolla CA). Specific details of tests used for each data set are given in the figure legends. For gene expression bar graphs by both Nanostring and qRT-PCR, technical replicates were averaged to produce biological replicates derived from individual animals. The heat maps of gene expression changes illustrate each technical replicate individually. Statistical analyses of changes in gene expression by Nanostring were assessed by multiple t-tests with a false discovery rate set at 2% using GraphPad Prism.

All authors had access to review the study data and have approved the final manuscript.

## Results

### The dsRNA mimetic Poly I:C alters morphology and survival of jejunal enteroids

**Fig 1:**
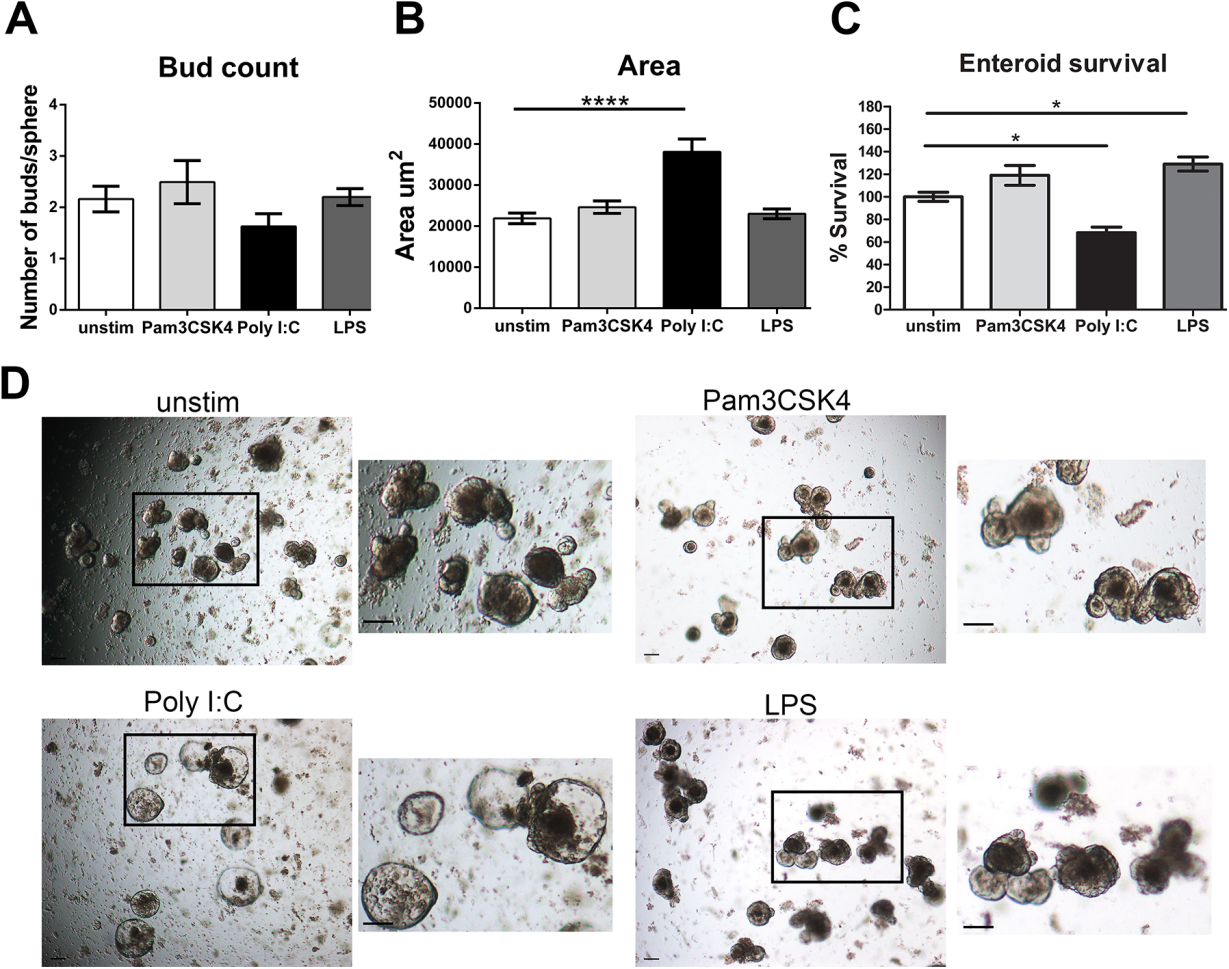
Growth morphology and survival is impacted by Poly I:C stimulation of enteroids. Crypts from the jejunum were plated in Matrigel for two days. Pam3CSK4 (400ng/mL), Poly I:C (40μg/mL) or LPS (13ug/mL) was added to the media on day 2 and day 4. Two days after the last addition of Poly I:C, (D6) images were obtained from at least four areas of interest of triplicate wells at multiple depths of focus. **A)** Buds on individual enteroids were counted using images at multiple depths of focus, n = 6 individual jejunal preparations. **B)** The surface area of the enteroids was measured using the free hand drawing tool in the NIS-Elements software, n = 6. **C)** The percentage of enteroids surviving stimulation to day 6 compared to unstimulated controls was calculated in triplicate wells, n = 3. **D)** Images of unstimulated and stimulated enteroids. Images were taken at 4X, scale bars represent 100μm. Bars represent mean ± SEM. Significance determined by One-way ANOVA, Dunnett’s multiple comparison test, * p<0.05, **** p<0.0001.

We began our analysis of the responsiveness of enteroids and colonoids by assessing the impact of a panel of microbial-derived stimuli. Jejunal crypts were isolated and plated in Matrigel for 2 days prior to the addition of Pam3CSK4, LPS or Poly I:C. At day 6 of culture, images were obtained at multiple focal depths. We found that stimulation of enteroids with Pam3CSK4 and LPS had little impact on either bud count or area of the enteroids. In contrast, Poly I:C significantly increased the surface area of enteroids; there was also a trend toward decreased bud count on individual enteroids (**Fig 1A, 1B and 1D**). Poly I:C also significantly decreased the survival of enteroids at day 6, while LPS increased the survival of enteroids (**Fig 1C**).

### Colonoid cultures have reduced budding in response to Poly I:C

Growth of colonoids from isolated crypts has not been a readily reproducible technique compared to the small intestine. After sacrifice, we washed the colonic tissue and placed it at 4°C overnight. This step allowed differentiated epithelial cells at the top of the crypts to dissociate from the bottom crypt cells, which in many cases had already formed into spheres upon isolation the next day. We also found that addition of 5μM of Chir99021, a WNT pathway activator and GSK3β inhibitor, throughout the duration of culture was necessary to maintain colonoid survival. Removal of Chir99021 at day 4/5 lead to colonoid death. Colonoid budding occurred at a later time point than that of enteroids and as such we analyzed colonoid cultures on day 8 compared to day 6 for enteroids.

**Fig 2:**
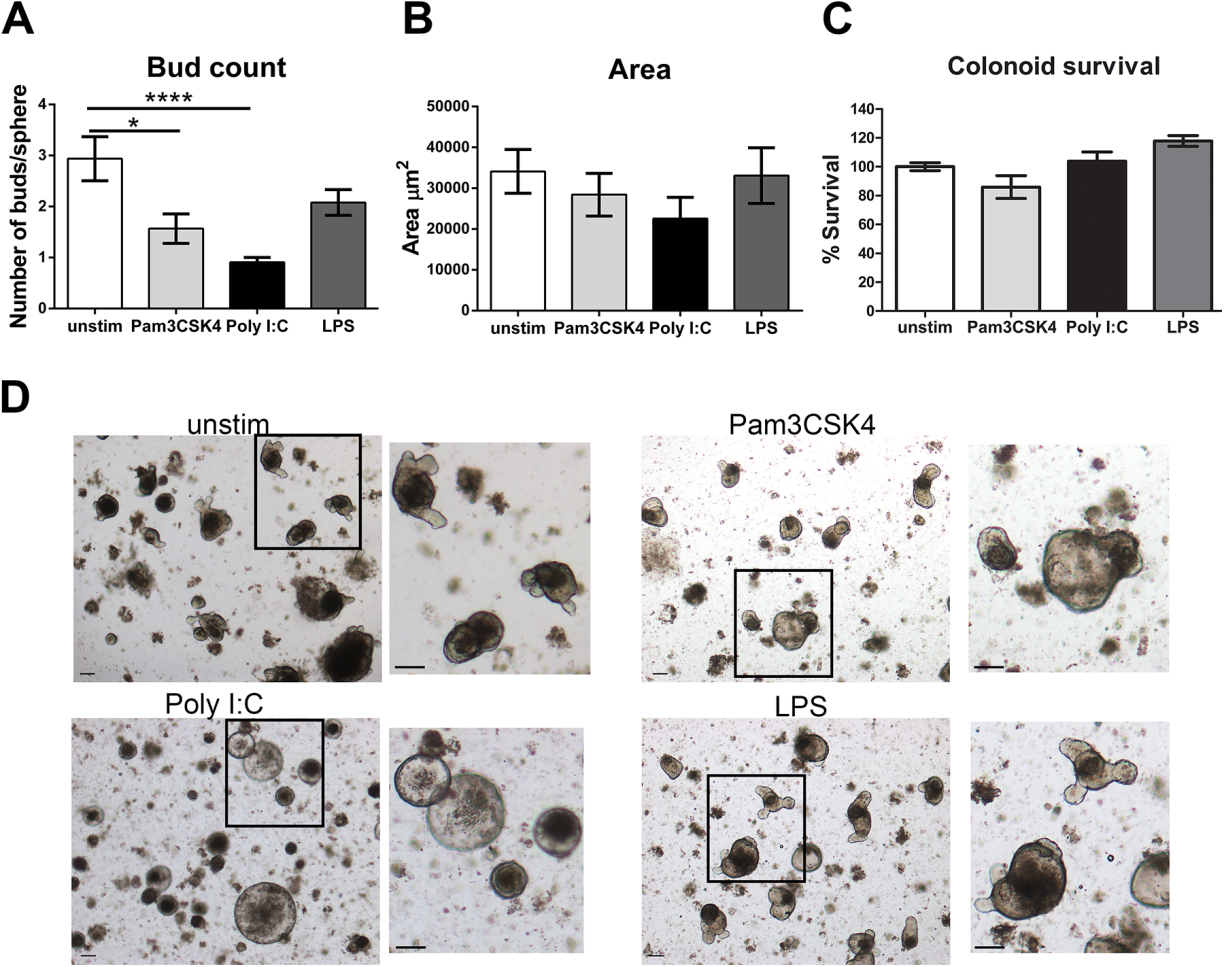
Colonoid budding is reduced in response to Poly I:C. Crypts from the entire colon were plated in Matrigel for two days. Pam3CSK4 (400ng/mL), Poly I:C (40μg/mL) or LPS (13ug/mL) was added to the media on day 2, 4 and 6. Media was changed on day 4. Two days after the last addition of Poly I:C images were obtained on day 8, n = 6 individual colon preparations. **A)** Bud count was determined on colonoids similar to enteroids on day 8 of culture, n = 6. **B)** The surface area of colonoids was determined as in the enteroids on day 8, n = 6. **C)** The percentage of colonoids surviving stimulation compared to unstimulated controls was determined at day 8, n = 3. **D)** Images of colonoid cultures were obtained at 4X, scale bars represent 100μm. Bars represent mean ± SEM. Significance determined by One-way ANOVA, Dunnett’s multiple comparison test, ** p<0.01, **** p<0.0001.

Colonoids (derived from proximal to distal colon, not including the cecum) were plated in Matrigel for 2 days prior to the addition of stimuli, similar to enteroids. We found that culture of colonoids in the presence of Pam3CSK4 decreased the bud count. Poly I:C also significantly decreased the bud count of colonoids and this effect was much more pronounced compared to that observed with Poly I:C stimulation in enteroids (**Fig 2A and 2D**). Conversely, the surface area of colonoids stimulated with Poly I:C was not increased, and actually tended to be decreased compared to unstimulated control cultures (**Fig 2B and 2D**). Survival of the colonoids in the presence of any of the stimuli was not impacted (**Fig 2C**). Therefore, stimulation of enteroids and colonoids with Poly I:C leads to a generally similar decrease in budding, but had dissimilar effects on surface area. Also, enteroids were more sensitive to Poly I:C stimulation having decreased survival, while survival was unaffected in colonoids. Pam3CSK4 and LPS had minimal impact on the morphology of enteroids and colonoids, and as such we focused the remainder of our investigations on the response to Poly I:C.

### Rates of proliferation in enteroids and colonoids are not affected by Poly I:C stimulation

**Fig 3:**
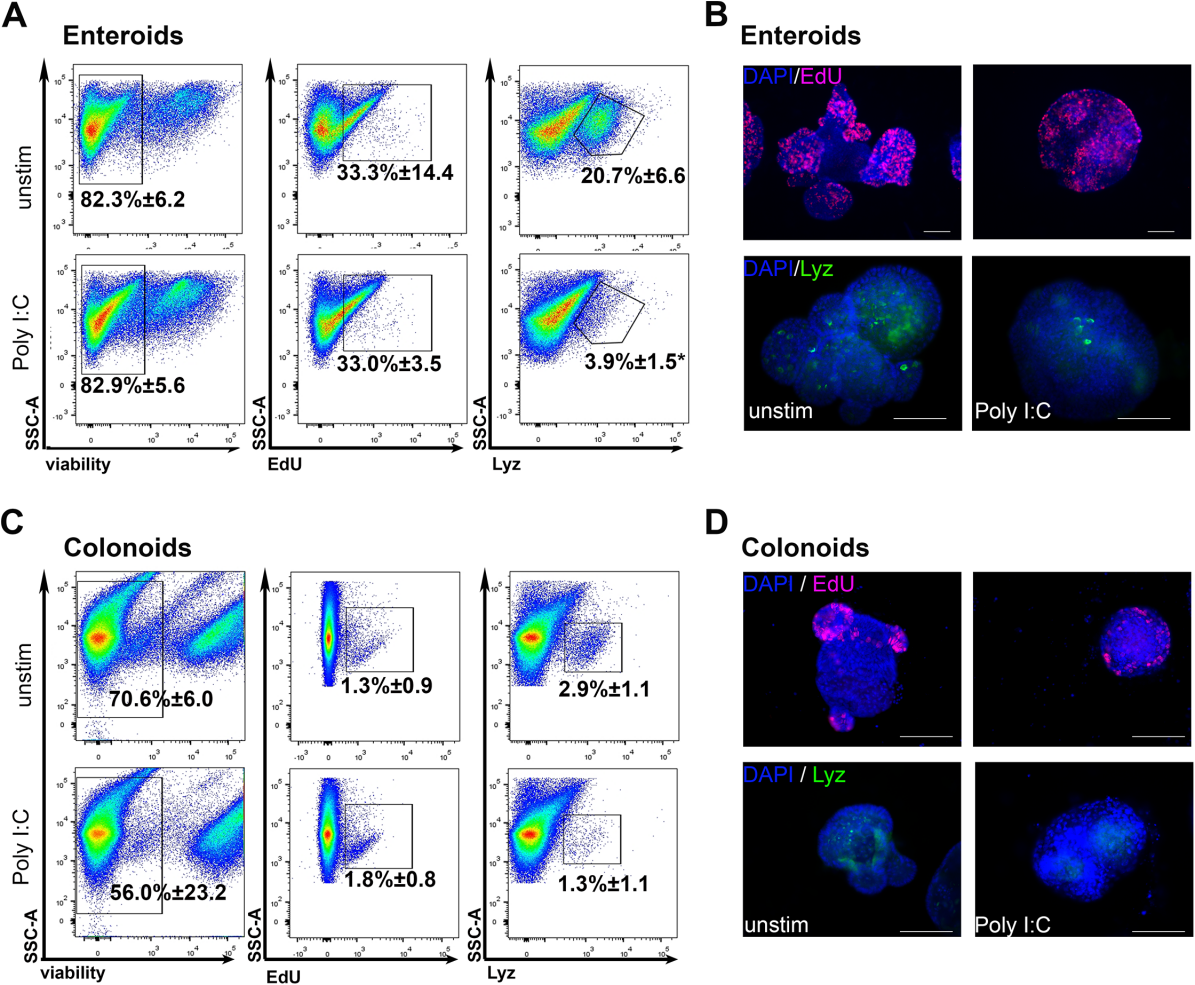
Poly I:C stimulation significantly decreased the number of lysozyme positive cells in enteroids. Enteroids and colonoids were grown and stimulated as in Figs 1 and 2. **A,C)** At the end of culture (day 6 for enteroids and day 8 for colonoids) organoids were pulsed with EdU for 50 min at 37°C and then rendered to single cells and stained for flow cytometric analysis. **B,D)** Additional organoids were assessed for EdU incorporation or stained for lysozyme using immunofluorescence. Scale bars represent 100μm. Values in flow plots are mean ±SEM of percentage of events falling in the indicated gates. Significance determined by unpaired Student’s t-test. *p<0.05, n = 3 independent preparations.

To determine if increased proliferation was responsible for the enlarged enteroid surface area, we assessed proliferation following a short pulse of EdU on day 6 of culture. Enteroids were pulsed with EdU and then digested out of the Matrigel and rendered to a single cell suspension. EdU incorporation was assessed by flow cytometry (**Fig 3A and 3B**). The percentage of cells that were EdU positive following Poly I:C stimulation was equal to the percentage in the unstimulated cultures. Similar to the enteroids, the percentage of proliferating cells in colonoids at the end of 8 days of culture was not altered by the addition of Poly I:C (**Fig 3C and 3D**). Therefore, the Poly I:C induced increase in enteroid surface area was not due to changes in proliferation rates.

### Lysozyme (Lyz) positive cells are decreased in response to Poly I:C stimulation

Since stimulation with Poly I:C altered the morphology of enteroids and colonoids, we next assessed its specific impact on subsets of epithelial cells. Lysozyme is an anti-microbial protein that is a major component of the granules found in small intestinal Paneth cells and a minor component of secretory cells in the colon. Release of the contents of Paneth cell granules has been shown to occur in response to muscarinic acetylcholine receptor stimulation, bacterial recognition [23] and immune cell-derived IFNγ [24]. In unstimulated enteroids, approximately 20% of cells were found to be Lyz positive (20.7% ± 6.6). Following culture in Poly I:C, this percentage was significantly decreased by five-fold (3.9% ± 1.5, p = 0.013) (**Fig 3A and 3B**). As expected, the number of Lyz positive cells in colonoids was lower than that found in enteroids (20.7% ± 6.6 in enteroids compared to 2.9% ± 1.1 in colonoids). Stimulation of colonoids with Poly I:C decreased the number of Lyz positive cells (1.3% ± 1.1) by over half, although this was not statistically significant p = 0.15) (**Fig 3C and 3D**). Thus, Poly I:C reduces the percentage of cells harboring high levels of lysozyme in enteroids and colonoids, likely reflecting degranulation of Paneth cells in the enteroids and secretory cells in the colonoids in response to Poly I:C stimulation.

### Enteroids down-regulate stem cell marker expression following Poly I:C stimulation

**Fig 4:**
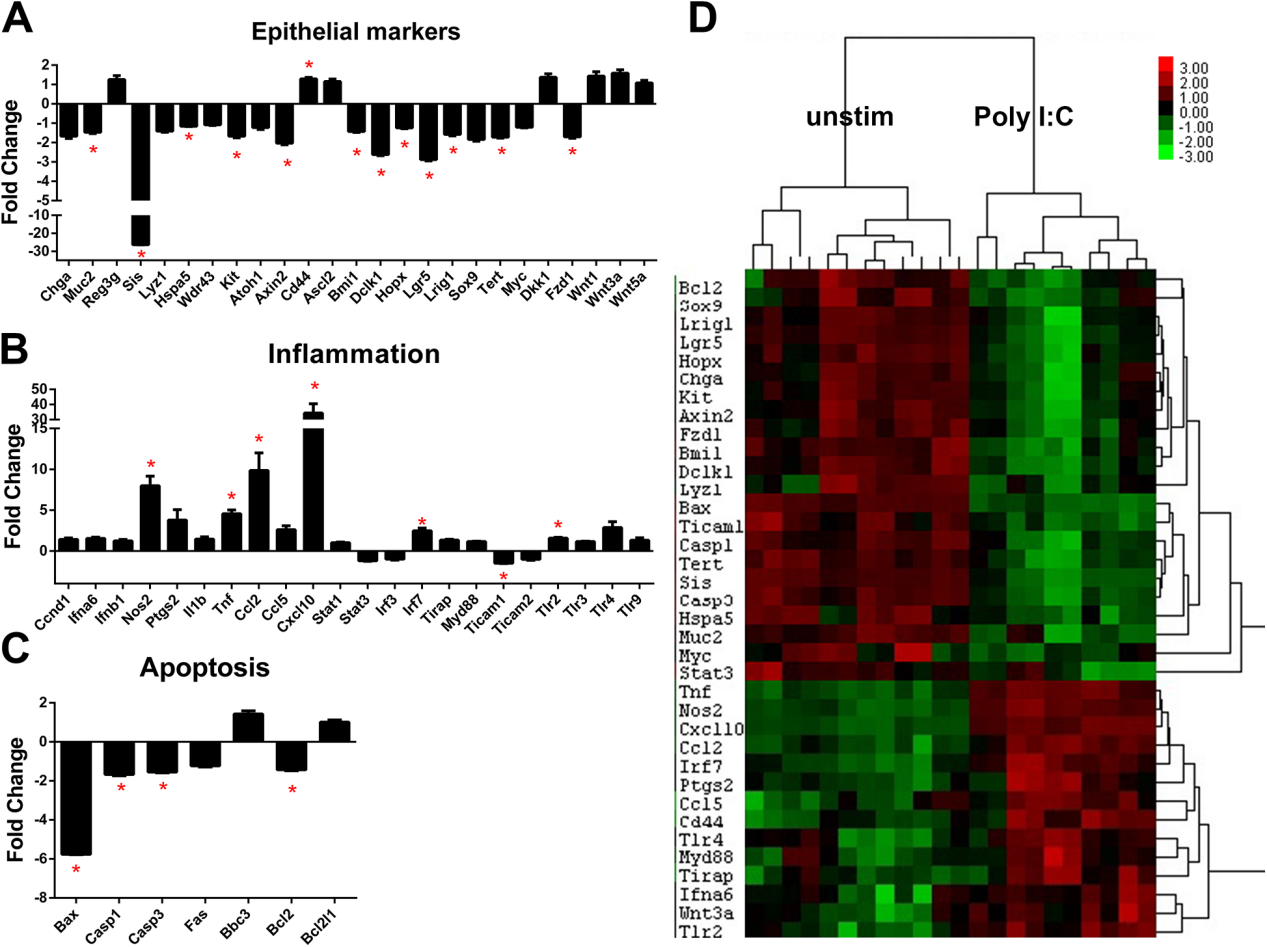
Enteroid gene expression changes induced by Poly I:C stimulation. Enteroids were grown and stimulated as in Fig 1. Gene expression levels were determined using Nanostring probe-based analysis of isolated RNA. **A)** Fold change in normalized gene expression are grouped by category. Bars represent mean ±SEM compared to unstimulated samples. n = 5 (Poly I:C stimulation)/6 (unstim) duplicate samples. Discoveries (*) determined by false discovery rate following multiple t-test analysis. **B)** Heat map of the normalized probe counts of unstimulated and Poly I:C stimulated enteroids of each technical replicate. Samples and genes were clustered by Pearson correlation.

We next asked what changes in gene expression were induced in response to Poly I:C stimulation. To answer this question, we interrogated a panel of genes representing epithelial differentiation, inflammation, and apoptosis using Nanostring probe-based RNA quantification (see **S1 Table** for complete panel of genes). Stem cell markers including: Bmi1, Dclk1, Hopx, Lgr5, Lrig1 and Tert were all significantly decreased following Poly I:C exposure compared to unstimulated enteroids (**Fig 4A** and **S3 Table**). Markers of absorptive (Sis) and goblet cell (Muc2) lineages were also significantly decreased in enteroids stimulated by Poly I:C. In contrast to the loss of Lyz+ cells that we observed by flow cytometry (Fig 3), we found no significant decrease in expression of Lyz1 in response to Poly I:C consistent with our hypothesis that there is continual release of lysozyme-containing granules after their formation due to repeated Poly I:C stimulation.

With respect to inflammatory gene expression, numerous inflammatory genes were found to be significantly increased in enteroids following culture in Poly I:C. We noted inflammatory genes including Nos2 (iNOS), Tnf, Cxcl10 (IP-10) and Irf7 were significantly upregulated while Ticam1 (TRIF) was significantly decreased (**Fig 4B**). Increases in IP-10 [12] and iNOS [25] have previously been described in colon cell line response to rotavirus infection. Poly I:C stimulated enteroids had decreased expression of pro-apoptotic genes Bax, Casp1 and Casp3, but also a significant decrease in the pro-survival gene Bcl2 (**Fig 4C**). We confirmed the Nanostring results with qRT-PCR analysis of a selection of genes (**S1 Fig**). Distinct clustering was detectable in the unstimulated versus Poly I:C stimulated enteroids when plotted in a heat map (**Fig 4D**). Genes with decreased expression after Poly I:C stimulation were mainly epithelial markers of differentiation and apoptosis-related genes, while inflammatory genes were clustered as increased in Poly I:C treated samples. Thus, when stimulated with Poly I:C enteroids decrease expression of differentiation and stem cell markers and activate an inflammatory response.

### Colonoids have a muted response to Poly I:C stimulation compared with enteroids

**Fig 5:**
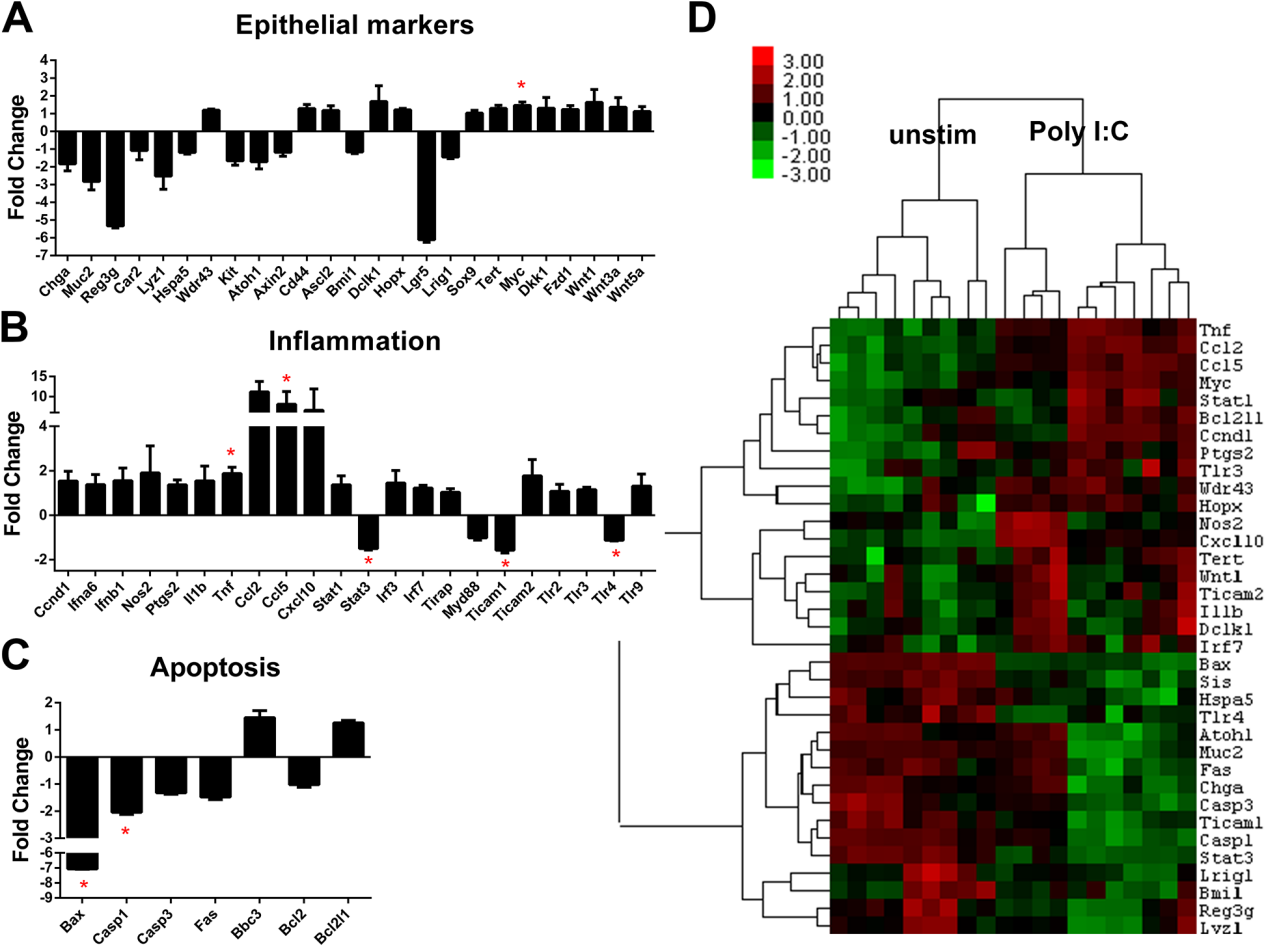
Colonoid gene expression changes induced by Poly I:C stimulation. Colonoids were grown and stimulated as in Fig 2. Gene expression levels were determined using Nanostring probe-based analysis of isolated RNA. **A)** Fold change in normalized gene expression are grouped by category. Bars represent mean ±SEM compared to unstimulated samples. n = 6 (Poly I:C stimulation)/5 (unstim) duplicate samples. Discoveries (*) determined by false discovery rate following multiple t-test analysis. **B)** Heat map of the normalized probe counts of unstimulated and Poly I:C stimulated colonoids of each technical replicate. Samples and genes were clustered by Pearson correlation.

When colonoids were examined for changes in gene expression induced by Poly I:C stimulation fewer significant changes were observed compared to enteroids (**S4 Table**). Stem cell markers, including Bmi1, Dclk1, Lrig1, Sox9 and Hopx were not impacted by Poly I:C in colonoids (**Fig 5A**). However, Lgr5 expression was decreased by six-fold in Poly I:C stimulated colonoids but this was not statistically significant. Markers of epithelial subset lineages were also not as markedly impacted in colonoids compared to enteroids.

Similar to enteroids, stimulation of colonoids significantly increased inflammation-associated genes Tnf and Ccl5 (**Fig 5B**) and significantly decreased expression of Ticam1 as well as the apoptosis activators Bax and Casp1 (**Fig 5C**). Clustering of similarly regulated genes in stimulated colonoids was not as distinct as demonstrated in the enteroids. However, upregulated genes were generally inflammation-associated and decreased genes were differentiation markers and apoptosis-related (**Fig 5D**).

**Fig 6:**
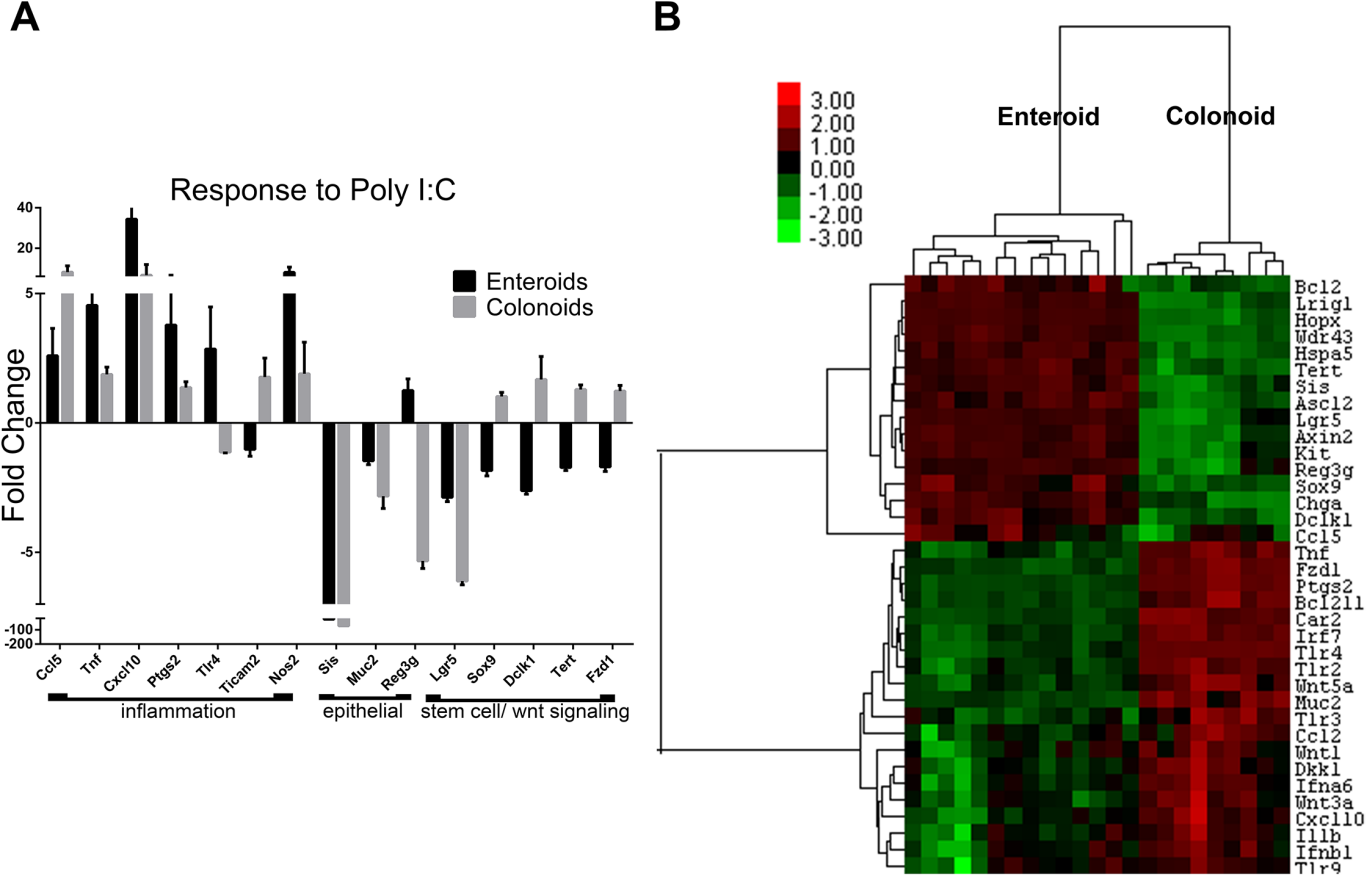
Differential response of enteroids and colonoids to Poly I:C stimulation. Fold change in gene expression of enteroids stimulated with Poly I:C and colonoids stimulated with Poly I:C were compared for differences in either magnitude of response or changes in the direction of response. **A)** Genes with altered direction or magnitude of response are graphed. **B)** Gene expression in unstimulated enteroids and colonoids was compared using enteroid expression as baseline. Samples and genes were clustered by Pearson correlation.

In examining the gene expression data, we wanted to highlight any regional differences in the responsiveness between enteroids and colonoids to Poly I:C stimulation. Therefore, we plotted the fold change in gene expression in enteroids and colonoids in response to Poly I:C stimulation. Most genes were altered in direction and magnitude to a similar degree in enteroids and in colonoids (**S2 Fig**). However, there were a few exceptions (**Fig 6A**). Inflammatory genes including Cxcl10 (IP-10), Ptgs2 (COX-2), Tlr4, Tnf and Nos2 (iNOS) were all increased to a greater extent in enteroids compared to colonoids. Conversely, Ccl5 was increased to a greater extent in colonoids. The antimicrobial gene Reg3g and Muc2 were decreased to a greater extent in Poly I:C stimulated colonoids compared to enteroids. Finally, stem cell markers and Wnt signaling molecules including Sox9, Dclk1, Tert and Fzd1 were decreased in enteroids but unchanged in colonoids. In contrast, reduction of Lgr5 was greatest in colonoids compared to enteroids. These data indicate that while most of the Poly I:C responses are shared between enteroids and colonoids, there are certain gene expression changes which are distinctive to each location.

### Enteroids and colonoids have different molecular signatures at baseline

To examine potential reasons for the differences observed in response of enteroids and colonoids to Poly I:C stimulation, we compared the baseline gene expression from these two sites. Generating a heat map of the gene expression levels between the two unstimulated sites revealed stark differences between cultures generated from the jejunum versus the colon (**Fig 6B and S5 Table**). As expected, region specific markers like Sis and Reg3g were increased in the enteroids compared to colonoids and conversely Car2 was increased in colonoids compared to enteroids. Interestingly, stem cell markers (Sox9, Dclk1, Lrig1, Hopx, Lgr5) were increased in the enteroids compared to colonoids. Chemokines Ccl2 and Cxcl10 and toll-like receptors Tlr2, Tlr3, Tlr4 (**S1B Fig**) and Tlr9 were all increased in the colonoids, but the TLR adaptor proteins MyD88 and Ticam1 were decreased (**S1C Fig**). Wnts 1, 3a and 5a were increased in colonoids compared to enteroids and Wnt pathway genes like Fzd1 and Dkk1 were also increased in comparison to enteroids. These data suggest that there are inherent differences in the levels of known stem cell markers between the small intestine and colon and these may underlie differences in growth kinetics, morphology, and risk of malignancy.

### Ticam1 mediates the response of enteroids to Poly I:C but not the colonoid response

**Fig 7:**
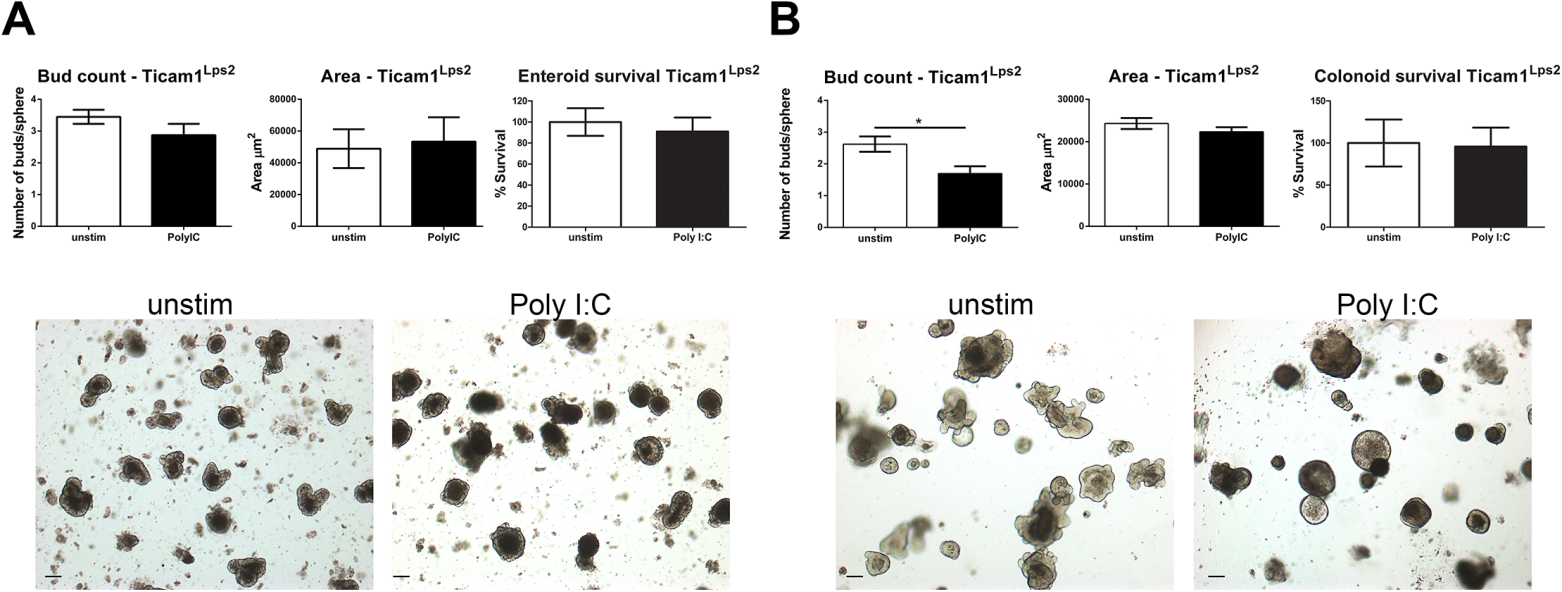
Loss of Ticam1 signaling blocks Poly I:C induced increases in enteroid area. Enteroids and colonoids were grown from Ticam1^Lps2^ jejunum and colon. **A)** Enteroids cultured in Poly I:C were assessed for buds, area and survival percentage as in Fig 1, n = 6 individual preparations. **B)** Colonoids were cultured in Poly I:C and assessed for buds, area and survival percentage as in Fig 2, n = 6. Bars represent mean ± SEM. Images were obtained at 4X, scale bars represent 100μm. Significance determined by Student’s t-test, *p<0.05.

Poly I:C induced TLR3 signaling is mediated by the adaptor protein Toll/IL-1R domain-containing adapter-inducing interferon-β (TRIF). However, other cytoplasmic viral sensors including retinoic acid-inducible gene 1 (RIG1) and melanoma differentiation-associated gene 5 (MDA5) also recognize Poly I:C and initiate inflammatory signaling. To determine which receptor was mediating the response of enteroids and colonoids to Poly I:C we cultured enteroids and colonoids from Ticam1^Lps2/Lps2^ mice that have a mutated Ticam1 gene which prevents signal transduction through TLR3. Enteroids were grown and stimulated as in Fig 1, and the area, bud count and survival analyzed (**Fig 7A**). Ticam1^Lps2/Lps2^ enteroids grown in the presence of Poly I:C were the same area, and had the same number of buds and the same survival rates as unstimulated enteroids indicating that the altered morphology of enteroids induced by Poly I:C is mediated through TLR3 signaling. Conversely, when colonoids from Ticam1^Lps2/Lps2^ mice were grown in Poly I:C they continued to demonstrate decreased budding, although the reduction was not as marked as that seen in wild-type colonoids (**Fig 7B**). These data suggest that TLR3 signaling through TRIF is responsible for the phenotypic responses of enteroids to dsRNA. In the colon, TLR3 signaling only partially explains this phenotype.

## Discussion

The goals of our study were to understand epithelial responses to PAMPs present in the intestine, and also to address fundamental differences in the sensitivity of small intestinal and colonic epithelial cells to immune-mediated pathology. We reasoned that the response to a mimetic of a dsRNA virus, which often have their most profound effects on the small bowel, would differ in cultures derived from the small intestine compared with those of the colon. We have also employed a variation of previously published methods to consistently culture colonoids that bud similarly to enteroids without the need for a feeder layer. Our study demonstrates that Poly I:C strikingly alters morphology—both enteroids and colonoids fail to show budding of crypts when stimulated with Poly I:C. This very dramatic response in the morphology of the organoids is also specific to Poly I:C since neither LPS (TLR4 ligand) nor Pam3CSK4 (TLR1/2 ligand), induces these changes. We also found at a molecular level this change in morphology is accompanied by a decrease in stem cell and epithelial lineage marker expression and an increase in inflammatory gene expression especially in enteroids. We have outlined specific differences in baseline gene expression and response to stimulation between in enteroids and colonoids which may underlie their diverse susceptibility to viral pathology *in vivo*.

In this study, we investigated the response of enteroids and colonoids to a panel of TLR ligands, and found no morphological response to either Pam3CSK4 or LPS. We did not examine protein expression of the TLRs in unstimulated or stimulated enteroids, and lack of response to Pam3CSK4 and LPS may be attributable to low protein expression levels. Only stimulation with the mimetic of double stranded RNA and TLR3 ligand Poly I:C altered the phenotype of the cultures. An important example of a virus with a dsRNA genome is the intestinal pathogen rotavirus [26, 27]. Rotavirus has been shown to induce signaling through TLR3 [28, 29], and villous pathology similar to that observed in rotavirus infection can be induced by intraluminal injection of Poly I:C [30]. TLR3 is expressed at high levels in epithelial cells [9]–even higher than the lamina propria or mesenteric lymph nodes [31]. This suggests that epithelial cells may be the most important mediator of the response to this pathogen-associated molecular pattern (PAMP) in the intestine. Beyond viral-derived products, recent studies have demonstrated that bacterial RNAs [32] as well as endogenous RNAs [33] can also form dsRNAs that can be recognized by TLR3. Depending on the cell type, the response to dsRNA varies from apoptosis [34], to necrosis [35, 36] or induction of interferon β [32]. Poly I:C may also be an important transducer of stem-ness as it was found to induce epigenetic changes in chromatin which were necessary for efficient re-programming of pluripotent stem cells [37]. Therefore, Poly I:C is a useful ligand as it may mimic the response to a variety of different pathogen or damage-related stimuli and the human pathogen rotavirus.

Intestinal recognition of viral PAMPs *in vivo* is mediated by both immune cells and epithelial cells. Poly I:C is a dsRNA mimetic that is recognized by TLR3, but has also been shown to activate other cytoplasmic viral sensors including retinoic acid-inducible gene 1 (RIG1) and melanoma differentiation-associated gene 5 (MDA5). In our study we were able to determine that the observed reduction in budding and increase in surface area in enteroids following Poly I:C stimulation was dependent on TRIF signaling (**Fig 7**). The increase in surface area was not due to transient swelling [38], as area was measured two days following the final addition of Poly I:C to the culture supernatants.

Conversely, the loss of TRIF in colonoids did not significantly rescue the budding defect. Differences in pathways mediating responses to the same stimulation between the small and large intestine has previously been demonstrated. Sommer and Backhed recently described differences in the regulation of Duox2 expression between the ileum and the colon [39]. They found that Duox2 expression was increased in the ileum through a TRIF dependent mechanism, while Duox2 upregulation in the colon was driven by MyD88. Our data indicate that the response to dsRNA in the colon may be mediated by functionally redundant pathways.

The most pronounced feature of the response to Poly I:C was the deficit in budding (**Figs 1A and 1D, 2A and 2D**). Crypt fission *in vivo* and budding *in vitro* have been modeled using biomechanical theory. Early theories posited that crypts underwent fission due to an increased number of daughter stem cells in the crypt, which put pressure on the crypt area and led to buckling out of the crypt region to produce a new crypt [40, 41]. However, a recent report assessing the dynamics of crypt budding *in vitro* has proposed that budding is driven by differences in the fluidity and resistance to deformation of the two types of cells in the crypt base, stem cells and Paneth cells—specifically that stem cells are more elastic and easier to deform than Paneth cells filled with rigid cytosolic granules. Their model suggests that stem cells are pushed out of the plane of the crypt when they proliferate when surrounded by rigid Paneth cells which allows for the creation of a bud [42, 43]. Our data demonstrate that Poly I:C reduces the number of Lyz+ cells in enteroids and colonoids. Thus, we speculate that the decrease in budding is due to the loss of the rigid lysozyme containing granules in Paneth cells in enteroids and secretory crypt cells in the colonoids required for the creation of new buds *in vitro*. This requirement for Paneth cells to induce budding and fission *in vitro* may not be required *in vivo* as loss of Paneth cells did not impact the ability of small intestinal crypts to heal following radiation injury [44].

*In vivo*, the small intestine and colonic epithelium differ in their topography, rates of growth, and susceptibility to malignancy. They also have vastly different densities of associated microbiota with the colon bearing the greater burden. The baseline gene expression between enteroids and colonoids differs significantly in levels of inflammatory and stem cell-associated genes (**Fig 6B**). These differences highlight that the colonic epithelium has a higher resting inflammatory tone than the jejunum, presumably in response to the greater microbial burden of the colon. We observed that enteroids have increased gene expression of numerous stem cell markers compared to colonoids including: Lgr5, Sox9, Dclk1, Hopx and Lrig1 (**Fig 6B** and **S5 Table**). It remains to be addressed whether there are fewer stem cells per colonoid, or if colon stem cells just express a decreased quantity of the markers compared to enteroids.

Despite overall similarity between responses elicited by enteroids and colonoids to Poly I:C, we noted several inflammatory genes that were increased to a greater extent in enteroids compared to colonoids including: Tnf, Cxcl10 (IP-10), Ptgs2 (COX-2) and Tlr4 (**Fig 6A**). This is interesting considering that the baseline expression of most of those genes was higher in colonoids compared to the enteroids. On the other hand, the gene expression levels of TLR adaptors MyD88 and Ticam1 were actually reduced in the colonoids compared to the enteroids (**Fig 6B**). This may be an adaptive feedback mechanism to control responsiveness to TLR ligands which are present at a much higher concentration in the colon than in the small intestine and may contribute to the decreased induction of inflammatory genes in response to Poly I:C in the colonoids.

A recent comparison of human intestinal cells from small intestine and colon expanded on a feeder layer prior to embedding in Matrigel noted differences in the expression of stem cell markers and an increase in Wnt pathway genes in the colonoids compared to those from the enteroids which is consistent with our observations in murine enteroids and colonoids grown from crypt units [45]. The human study noted that organoids derived from the large intestine did not produce buds, and we suggest that this is due to insufficient exogenous Wnt signaling in their culture conditions. As Wnt signaling is required for Paneth cell formation, insufficient levels of Wnt signaling may sub-optimally promote the differentiation of Paneth cells and goblet-like cells (colon Paneth equivalents) from progenitors. Chir99021 is a GSK3β inhibitor and acts to amplify Wnt/β-catenin signaling. In our culture system, addition of 5uM Chir99021 to colon cultures (twice as much as in enteroid cultures) allowed the generation of buds in the colonoids (**Fig 2**).

Poly I:C reduced the gene expression of stem cell markers including Lgr5 and Lrig1, epithelial lineage markers Sis and Muc2 (**Fig 4**), and the number of lysozyme positive cells in enteroids (**Fig 3A**). Conversely, inflammatory genes like Nos2, Tnf and several chemokines were increased. We propose that as a protective mechanism in response to Poly I:C stimulation, small intestinal epithelial cells divert from normal epithelial function of absorption and digestion to instead produce inflammatory molecules to assist in limiting the dissemination of virus. This response was blunted in colonoids, where decreases in differentiation markers like Car2 and Muc2 were non-significant (**Fig 5**) as was the reduction in lysozyme positive cells (**Fig 3C**). As colonoids utilize a different signaling pathway to respond to Poly I:C than enteroids, the dichotomy in responses between enteroids and colonoids is more understandable. Differences in the usage of microbial recognition signaling pathways may explain the differences in sensitivity of the colon to virus-mediated pathology.

In summary, our study describes the response of small intestinal and colonic crypt cultures to a mimetic of dsRNA. The response is characterized by upregulation of inflammatory genes and decreases in gene expression of stem cell and differentiation markers in enteroids. Colonoids demonstrate a much less robust inflammatory response to Poly I:C despite higher baseline expression of PRRs and inflammatory genes compared to enteroids. This is accompanied by a change in the proportion of cells containing lysozyme and a decrease in crypt fission. We also provide evidence that there are location specific gene expression patterns and responses to PRR stimulation, which may underlie differences in susceptibility to viral infection along the intestinal tract.

## Supporting Information

S1 Fig Gene expression changes by qRT-PCR. Gene expression changes in enteroids and colonoids stimulated as in Figs 1 and 2 were validated using qRT-PCR, n = 5/6 independent preparations. Bars represent mean ± SEM. Significance determined by student’s t-test compared to unstimulated, **** p<0.0001, *** p<0.001, ** p<0.01, * p<0.05.
(TIF)

S2 Fig Comparison of gene expression changes in Poly I:C stimulated enteroids and colonoids.
The fold change of gene expression induced by Poly I:C stimulation as determined by Nanostring probe based analysis was plotted for enteroids and colonoids. Bars represent mean ± SEM.
(TIF)

S1 Table Details of probes for Nanostring analysis. (PDF)

S2 Table Primers for qRT-PCR analysis. (PDF)

S3 Table Significantly altered gene expression of enteroids stimulated with Poly I:C. (PDF)

S4 Table Significantly altered gene expression of colonoids stimulated with Poly I:C. (PDF)

S5 Table Significantly altered gene expression between enteroids and colonoids at baseline. (PDF)

## Abbreviations

TLR: Toll-like receptor
Poly I:C: polyinosinic:polycytidylic acid
TRIF: TIR-domain-containing adapter-inducing interferon-β
Ticam1: toll-like receptor adaptor molecule 1
PAMP: pathogen-associated molecular patter
