# Supplementary figures and images for: The Viral Mimetic Polyinosinic:Polycytidylic Acid Alters the Growth Characteristics of Small Intestinal and Colonic Crypt Cultures
Source: PLoS One. 2015 Sep 28;10(9):e0138531. doi: 10.1371/journal.pone.0138531 (PMC4587363; doi:10.1371/journal.pone.0138531)

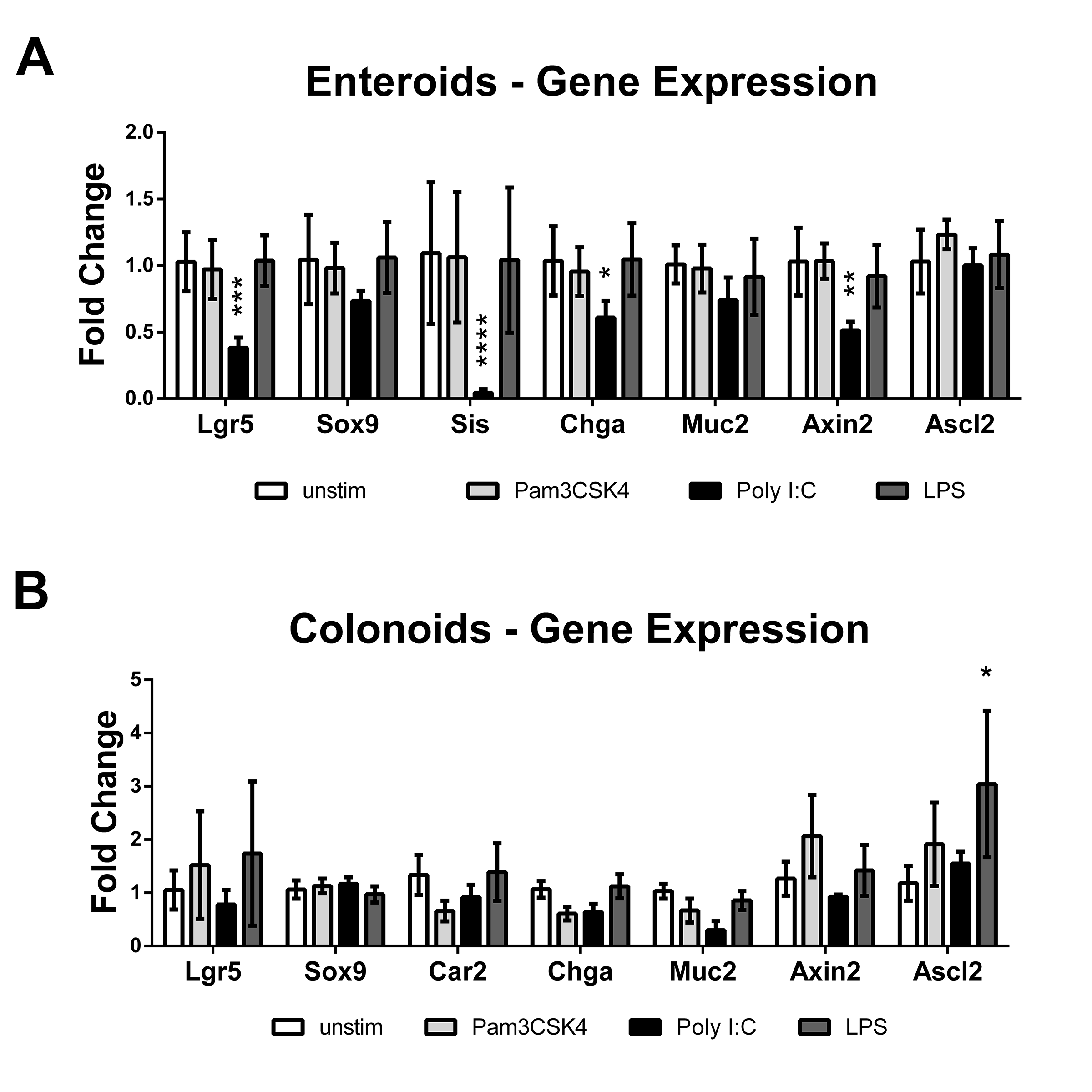

Supplement: S1 Fig — Gene expression changes in enteroids and colonoids stimulated as in Figs 1 and 2 were validated using qRT-PCR, n = 5/6 independent preparations. Bars represent mean ± SEM. Significance determined by student’s t-test compared to unstimulated, **** p<0.0001, *** p<0.001, ** p<0.01, * p<0.05. (TIF) [file pone.0138531.s001.tif]

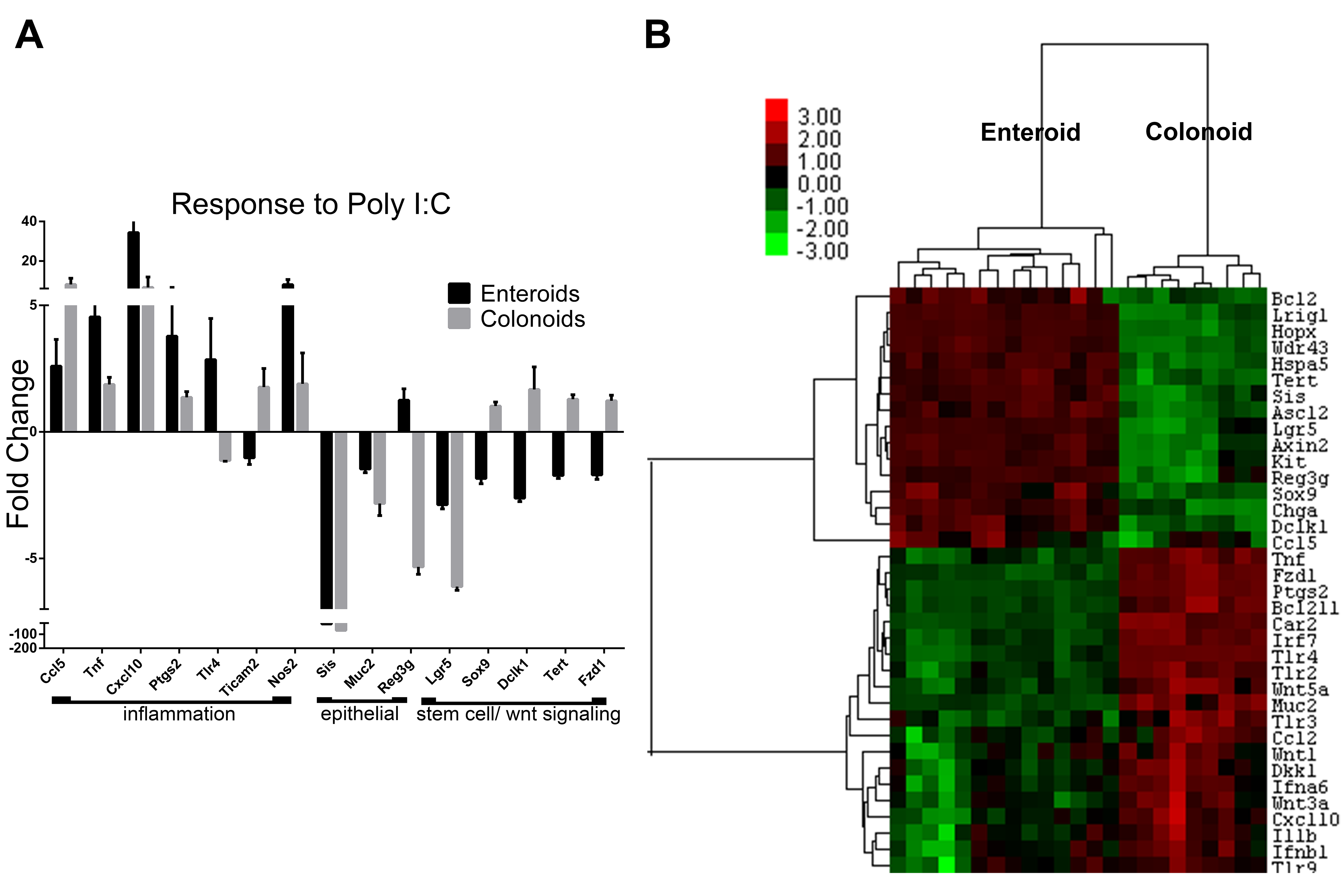

Supplement: S2 Fig — The fold change of gene expression induced by Poly I:C stimulation as determined by Nanostring probe based analysis was plotted for enteroids and colonoids. Bars represent mean ± SEM. (TIF) [file pone.0138531.s002.tif]
