# Supplementary material for: The Viral Mimetic Polyinosinic:Polycytidylic Acid Alters the Growth Characteristics of Small Intestinal and Colonic Crypt Cultures
Source: PLoS One. 2015 Sep 28;10(9):e0138531. doi: 10.1371/journal.pone.0138531 (PMC4587363; doi:10.1371/journal.pone.0138531)
Supplement: S1 Table — (PDF) [file pone.0138531.s003.pdf]

Table S1. Probe design for Nanostring analysis

Gene	Accession	Target Region	# isoforms hit by probe	# isoforms not hit by probe
Actb	NM_007393.3	1195-1294	1	0
Ascl2	NM_008554.2	346-445	2	0
Atoh1	NM_007500.4	1607-1706	1	0
Axin2	NM_015732.4	1121-1220	5	0
Bax	NM_007527.3	736-835	2	0
Bbc3	NM_133234.2	1831-1930	1	0
Bcl2	NM_009741.3	1845-1944	2	0
Bcl2l1	NM_009743.4	201-300	7	0
Bmi1	NM_007552.4	3355-3454	3	0
Car2	NM_009801.4	1175-1274	2	0
Casp1	NM_009807.2	260-359	1	0
Casp3	NM_009810.2	631-730	2	0
Ccl2	NM_011333.3	416-515	1	0
Ccl5	NM_013653.1	166-265	1	0
Ccnd1	NM_007631.1	2001-2100	1	0
Cd44	NM_009851.2	3076-3175	10	0
Chga	NM_007693.1	931-1030	1	0
Cxcl10	NM_021274.1	116-215	1	0
Dclk1	NM_001111051.1	4406-4505	9	2
Dkk1	NM_010051.3	56-155	1	0
Fas	NM_007987.2	96-195	2	0
Fzd1	NM_021457.3	3447-3546	1	0
Gstp1	NM_013541.1	1-100	1	0
Gusb	NM_010368.1	1736-1835	3	0
Hopx	NM_175606.3	873-972	3	0
Hspa5	NM_022310.3	559-658	2	0
Ifna1	NM_010502.2	355-454	1	0
Ifnb1	NM_010510.1	336-435	1	0
Il1b	NM_008361.3	1121-1220	2	0
Irf3	NM_016849.3	1528-1627	2	0
Irf7	NM_016850.2	706-805	4	0
Kit	NM_001122733.1	4276-4375	2	0
Lgr5	NM_010195.2	1789-1888	1	0
Lrig1	NM_008377.2	4359-4458	4	0
Lyz1	NM_013590.4	1055-1154	1	0
Muc2	NM_023566.3	6765-6864	1	0
Myc	NM_010849.4	631-730	4	0
Myd88	NM_010851.2	1596-1695	1	0
Nos2	NM_010927.3	3716-3815	2	0
Ptgs2	NM_011198.3	676-775	1	0
Reg3g	NM_011260.1	420-519	1	0
Sis	NM_001081137.1	1315-1414	2	0
Sox9	NM_011448.4	3523-3622	1	0
Stat1	NM_009283.3	1591-1690	6	0
Stat3	NM_213659.2	2131-2230	3	0
Tbp	NM_013684.3	71-170	1	0
Tert	NM_009354.1	2163-2262	7	0
Ticam1	NM_174989.4	2712-2811	1	0
Ticam2	NM_173394.2	1244-1343	2	0
Tirap	NM_001177846.1	255-354	4	0
Tlr2	NM_011905.2	256-355	2	0
Tlr3	NM_126166.2	1166-1265	7	0
Tlr4	NM_021297.2	2511-2610	1	0
Tlr9	NM_031178.2	1802-1901	1	0
Tnf	NM_013693.1	1136-1235	2	0
Wdr43	NM_175639.1	523-622	2	0
Wnt1	NM_021279.4	2189-2288	2	0
Wnt3a	NM_009522.2	1280-1379	1	0
Wnt5a	NM_009524.2	3021-3120	6	0
