# Supplementary material for: The Viral Mimetic Polyinosinic:Polycytidylic Acid Alters the Growth Characteristics of Small Intestinal and Colonic Crypt Cultures
Source: PLoS One. 2015 Sep 28;10(9):e0138531. doi: 10.1371/journal.pone.0138531 (PMC4587363; doi:10.1371/journal.pone.0138531)
Supplement: S2 Table — (PDF) [file pone.0138531.s004.pdf]

Table S2. Primer sequences for qRT-PCR analysis

Gene	Left	Right	Amplicon length
Tbp (HK)	ccaatgactcctatgaccccta	cagccaagattcacggtagat	104
Lgr5	cttcactcggtgcagtgct	cagccagctaccaaataggtg	72
Sox9	cagcaagactctgggcaag	tccacgaagggtctcttctc	63
Muc2	gtggatgtgtgggacctga	ttgcagtcaaactcaaagtgc	64
Chga	cgatccagaaagatgatggtc	cggaagcctctgtctttcc	69
Axin2	gagagtgagcggcagagc	cggctgactcgttctcct	104
Ascl2	gggctagaagcaggtaggtcca	caggagctgcttgacttttcca	104
Sis	ccgtaatcgtttccggttc	tgtggcacttcgtatctcttgt	60
Car2	CAAGCACAACGGACCAGA	ATGAGCAGAGGCTGTAGG	122
Tlr2	ggggcttcacttctctgctt	agcatcctctgagatttgacg	111
Tlr3	gatacagggattgcacccata	tcccccaaaggagtacattaga	77
Tlr4	ggactctgatcatggcactg	ctgatccatgcattggtaggt	101
Mda5 (Ihif1)	CTTCCTGGATGTTCTGCGCCAA	CCGTGGGGAGGCAGATAATAAT	310
Rig-I (Ddx58)	CAAAAACCACCCATACAATCAG	CAAATGTGATGTGTACAGGAAG	502
