# Supplementary material for: The Viral Mimetic Polyinosinic:Polycytidylic Acid Alters the Growth Characteristics of Small Intestinal and Colonic Crypt Cultures
Source: PLoS One. 2015 Sep 28;10(9):e0138531. doi: 10.1371/journal.pone.0138531 (PMC4587363; doi:10.1371/journal.pone.0138531)
Supplement: S3 Table — (PDF) [file pone.0138531.s005.pdf]

Supplemental Table 3. Significantly altered gene expression in enteroids stimulated with Poly I:C

Gene name	Accession #	Fold Change	P value	t ratio	df
Cxcl10	NM_021274.1	34.36	0.0001831	6.0827	9
Ccl2	NM_011333.3	9.85	0.0014027	4.5416	9
Nos2	NM_010927.3	7.98	0.0000970	6.6199	9
Tnf	NM_013693.1	4.55	0.0000169	8.2723	9
Irf7	NM_016850.2	2.47	0.0015149	4.4880	9
Tlr2	NM_011905.2	1.57	0.0011874	4.6587	9
Cd44	NM_009851.2	1.29	0.0045178	3.7553	9
Hspa5	NM_022310.3	-1.16	0.0035447	3.9137	9
Hopx	NM_175606.3	-1.24	0.0022627	4.2130	9
Bcl2	NM_009741.3	-1.43	0.0060536	3.5669	9
Bmi1	NM_007552.4	-1.43	0.0006026	5.1507	9
Muc2	NM_023566.3	-1.46	0.0014677	4.5100	9
Ticam1	NM_174989.4	-1.51	0.0014550	4.5161	9
Casp3	NM_009810.2	-1.55	0.0000559	7.1131	9
Lrig1	NM_008377.2	-1.57	0.0032666	3.9676	9
Kit	NM_001122733.1	-1.67	0.0057486	3.6000	9
Casp1	NM_009807.2	-1.67	0.0009492	4.8183	9
Fzd1	NM_021457.3	-1.70	0.0072798	3.4496	9
Tert	NM_009354.1	-1.72	0.0000996	6.5973	9
Axin2	NM_015732.4	-2.03	0.0071416	3.4617	9
Dclk1	NM_001111051.1	-2.62	0.0015723	4.4622	9
Lgr5	NM_010195.2	-2.88	0.0001749	6.1204	9
Bax	NM_007527.3	-5.76	0.0000018	10.8731	9
Sis	NM_001081137.1	-26.32	0.0002759	5.7511	9
