# Supplementary material for: The Viral Mimetic Polyinosinic:Polycytidylic Acid Alters the Growth Characteristics of Small Intestinal and Colonic Crypt Cultures
Source: PLoS One. 2015 Sep 28;10(9):e0138531. doi: 10.1371/journal.pone.0138531 (PMC4587363; doi:10.1371/journal.pone.0138531)
Supplement: S4 Table — (PDF) [file pone.0138531.s006.pdf]

Supplemental Table 4. Significantly altered gene expression in colonoids stimulated with Poly I:C

Gene name	Accession #	Fold Change	P value	t ratio	df
Ccl5	NM_013653.1	8.11	0.0007960	4.9457	9
Tnf	NM_013693.1	1.87	0.0001850	6.0738	9
Myc	NM_010849.4	1.46	0.0028258	4.0638	9
Tlr4	NM_021297.2	-1.13	0.0006308	5.1167	9
Stat3	NM_213659.2	-1.50	0.0000452	7.3082	9
Ticam1	NM_174989.4	-1.57	0.0018028	4.3678	9
Casp1	NM_009807.2	-2.04	0.0008628	4.8872	9
Bax	NM_007527.3	-7.07	0.0000028	10.2820	9
