# Supplementary material for: The Viral Mimetic Polyinosinic:Polycytidylic Acid Alters the Growth Characteristics of Small Intestinal and Colonic Crypt Cultures
Source: PLoS One. 2015 Sep 28;10(9):e0138531. doi: 10.1371/journal.pone.0138531 (PMC4587363; doi:10.1371/journal.pone.0138531)
Supplement: S5 Table — (PDF) [file pone.0138531.s007.pdf]

Supplemental Table 5. Significantly altered gene expression in unstimulated colonoids compared to unstimulated enteroids

Gene name	Accession #	Fold Change	P value	t ratio	df
Ptgs2	NM_011198.3	84.51	0.0007695	4.9703	9
Car2	NM_009801.4	76.10	0.0034927	3.9234	9
Tlr4	NM_021297.2	29.43	0.0000000	54.7628	9
Fzd1	NM_021457.3	7.09	0.0000139	8.4734	9
Irf7	NM_016850.2	4.11	0.0000003	13.5272	9
Ccl2	NM_011333.3	3.04	0.0004728	5.3330	9
Muc2	NM_023566.3	2.80	0.0000658	6.9646	9
Tnf	NM_013693.1	2.80	0.0000041	9.8359	9
Wnt5a	NM_009524.2	2.36	0.0000238	7.9260	9
Tlr2	NM_011905.2	2.26	0.0000022	10.6044	9
Ifnb1	NM_010510.1	2.25	0.0028505	4.0580	9
Dkk1	NM_010051.3	2.15	0.0030671	4.0093	9
Bcl2l1	NM_009743.4	2.15	0.0000330	7.6070	9
Cxcl10	NM_021274.1	2.13	0.0009874	4.7900	9
Ifna6	NM_010502.2	2.10	0.0008942	4.8614	9
Wnt3a	NM_009522.2	2.10	0.0021140	4.2591	9
Wnt1	NM_021279.4	2.01	0.0022400	4.2198	9
Il1b	NM_008361.3	2.00	0.0016790	4.4168	9
Ccnd1	NM_007631.1	1.97	0.0049384	3.6977	9
Bbc3	NM_133234.2	1.81	0.0033289	3.9551	9
Tirap	NM_001177846.1	1.41	0.0054667	3.6322	9
Tlr3	NM_126166.2	1.30	0.0005326	5.2431	9
Myd88	NM_010851.2	-1.25	0.0046774	3.7328	9
Myc	NM_010849.4	-1.37	0.0080066	3.3894	9
Ticam1	NM_174989.4	-1.42	0.0052042	3.6639	9
Bcl2	NM_009741.3	-1.57	0.0000485	7.2445	9
Hspa5	NM_022310.3	-1.73	0.0000006	12.3675	9
Stat3	NM_213659.2	-2.41	0.0016100	4.4458	9
Tert	NM_009354.1	-2.41	0.0000020	10.7120	9
Wdr43	NM_175639.1	-2.77	0.0000001	15.1381	9
Sox9	NM_011448.4	-3.13	0.0014322	4.5271	9
Ascl2	NM_008554.2	-3.18	0.0000430	7.3562	9
Dclk1	NM_001111051.1	-3.50	0.0002603	5.7976	9
Kit	NM_001122733.1	-3.54	0.0000250	7.8777	9
Chga	NM_007693.1	-4.13	0.0000169	8.2767	9
Reg3g	NM_011260.1	-4.32	0.0001700	6.1436	9
Hopx	NM_175606.3	-4.34	0.0000000	26.6012	9
Lrig1	NM_008377.2	-5.14	0.0000001	14.7960	9
Lyz1	NM_013590.4	-9.30	0.0000143	8.4449	9
Axin2	NM_015732.4	-9.53	0.0000376	7.4825	9
Sis	NM_001081137.1	-9.96	0.0001908	6.0487	9
Lgr5	NM_010195.2	-11.81	0.0000016	11.0093	9
